# A Systematic Analysis of Additive Manufacturing Techniques in the Bioengineering of In Vitro Cardiovascular Models

**DOI:** 10.1007/s10439-023-03322-x

**Published:** 2023-07-19

**Authors:** Hemanth Ponnambalath Mohanadas, Vivek Nair, Akbar Abbas Doctor, Ahmad Athif Mohd Faudzi, Nick Tucker, Ahmad Fauzi Ismail, Seeram Ramakrishna, Syafiqah Saidin, Saravana Kumar Jaganathan

**Affiliations:** 1 Fresenius Medical Care North America, Concord, CA 94520 USA; 2 Computational Fluid Dynamics (CFD) Lab, Mechanical and Aerospace Engineering, University of Texas Arlington, Arlington, TX 76010 USA; 3 Faculty of Engineering, School of Electrical Engineering, Universiti Teknologi Malaysia, Johor Bahru, Malaysia; 4 Centre for Artificial Intelligence and Robotics, Universiti Teknologi Malaysia, Kuala Lumpur, Malaysia; 5 School of Engineering, College of Science, Brayford Pool, Lincoln, LN6 7TS UK; 6 School of Chemical and Energy Engineering, Advanced Membrane Technology Research Centre (AMTEC), Universiti Teknologi Malaysia, Skudai, Malaysia; 7 Department of Mechanical Engineering, Center for Nanofibers & Nanotechnology Initiative, National University of Singapore, Singapore, Singapore; 8 IJNUTM Cardiovascular Engineering Centre, Universiti Teknologi Malaysia, Johor Bahru, Malaysia

**Keywords:** Cardiovascular applications, Stents, Additive manufacturing, Heart models, Congenital heart disease

## Abstract

Additive Manufacturing is noted for ease of product customization and short production run cost-effectiveness. As our global population approaches 8 billion, additive manufacturing has a future in maintaining and improving average human life expectancy for the same reasons that it has advantaged general manufacturing. In recent years, additive manufacturing has been applied to tissue engineering, regenerative medicine, and drug delivery. Additive Manufacturing combined with tissue engineering and biocompatibility studies offers future opportunities for various complex cardiovascular implants and surgeries. This paper is a comprehensive overview of current technological advancements in additive manufacturing with potential for cardiovascular application. The current limitations and prospects of the technology for cardiovascular applications are explored and evaluated.

## Introduction

Industry 4.0—dubbed the fourth industrial revolution redefines the industrial and manufacturing world and also the healthcare, pharmaceutical, biotechnology, and medical device industries [1]. The combination of the technological advancements of modern manufacturing with revolutionary information technology will play a crucial role in both economic and technological advancement [2]. The fundamentals of industry 4.0 include virtualization, interoperability, decentralization, manufacturing, secure communication, and real-time capabilities. These fundamentals are driven by technological developments in the fields of blockchain, the Internet of Things (IoT), big data analytics, and Artificial Intelligence (AI) [3]. The introduction of the internet into the manufacturing world has made factories more intelligent, improved ergonomics, more adaptable, resource efficient, and has improved cost-effectiveness [4]. Examples of the underpinning technologies and systems are Enterprise Resource Planning (ERP), Radio Frequency Identification (RFID), digital platforms, and Additive Manufacturing (AM) [5].

It is likely that nine different technologies will define the future of industry and manufacturing: Simulation, Augmented Reality, Autonomous Robots, industrial “Internet of Things” Cloud, Cybersecurity, Additive Manufacturing, horizontal and vertical system integration, and Big Data Analytics [6]. Industrial revolution 4.0 fundamentals can be divided into two parts: the virtual environment (IoT, Big Data, Cloud Computing, etc.) and the physical environment which is represented by Autonomous and Additive Manufacturing [7]. The virtual environment collects information from the physical environment and disseminates it via computer networks and wireless connectivity (Internet of Things) and converts this information into statistical data for predictive computation and analysis (Big Data) thus illuminating relationships which helps in understanding and controlling supply chains, lean manufacturing, and business planning. Note that much of the processing and storage of this information and data occurs in the virtual world as cloud storage and computing [6].

The second part is the physical environment in which Additive Manufacturing (AM) plays a significant role in the transformation by means of its capability to produce customizable products with sophisticated attributes (materials, shapes, and dimensions) [8]. The early developments of the AM process were more suited to prototyping rather than production, and it has still not yet sufficient technical edge to replace conventional manufacturing techniques. However, certain technological advancements and the need for customized mass production is increasing the popularity of AM in the fields of healthcare and biomaterials [8].

AM is three-dimensional printing; the process converts the information contained in digital solid models piece by piece, surface by surface, and layer by layer into tool paths that allow the deposition of physical objects [9]. AM has undergone major advances in the last three decades. The era of AM started with creating models and prototypes [10]. The technology only later evolved into producing rapid and soft tooling (e.g., vacuum and silicone casting moulds) [11] and currently, the technology is used to produce customizable end-use parts and products [12].

Additive manufacturing has been gaining momentum and is emerging as a crucial adjunctive tool in medicine [13]. Additive manufacturing gained momentum in medicine with medical modeling for the preparation and training of clinicians for pre- and post-operative surgical planning and was later developed for the fabrication of hardware and medical device instruments [14]. Other applications in healthcare include anatomical personalization for prostheses, orthoses, and splints [15], manufacturing biomedical implants [16], pharmaceutical drug delivery systems [17], and bio-composite structures [18].

Cardiovascular diseases (CVD) are the leading global cause of death. CVD claimed 17.9 million lives in 2016 which accounts for 31% of deaths that year according to the World Health Organization [19]. The American Heart Association in 2018 tells us that 92.1 million American adults suffer from one or another form of cardiovascular illness [20]. Other developing and developed countries have also reported an increase in cardiovascular patient deaths. China reported in 2018 that around 290 million of its people were suffering from CVD [21]. 2017 European cardiovascular disease statistics show around 3.9 million deaths each year due to CVD which is approx. 45% of the total deaths in Europe [22].

There is a demonstrable need for concurrent evolution of cardiovascular science and medical device manufacturing to advance these emergent technologies from the conventional manufacturing age to industry 4.0 at a faster pace to meet the rising rate of the global need for cardiovascular medical interventions and related solutions for patient care. This literature review demonstrates that additive manufacturing is currently in the development phase in cardiovascular applications but is gaining rapid popularity among clinicians and researchers because it has the potential to offer economical, feasible, and most importantly patient-specific applications. This review article explores the use of additive manufacturing in the advancement of cardiovascular treatments and evaluates its current limitations and prospects.

## Literature Search Methods

Initially, a detailed study of the applications of additive manufacturing, 3D modeling, and 3D printing in cardiovascular procedures is performed. This was achieved by probing into scientific literature accessible on Google Scholar and PubMed databases, focusing on the technologies as applied to cardiovascular methodologies.

The search criteria were divided into three sections:

1. Keywords related to Manufacturing Technology (such as Additive manufactu*, 3D print*, 3-dimensional),
2. Terminology for Anatomical Structures, Organs, and Tissues (including aort*, mitral, congenital heart disease, tumors, valve, cardio*, blood, vascular, surg*),
3. Application terminology (like stimulation, training, procedural planning, personalized aortic device, functional flow model, education, stent, grafts, blood compatibility, biomaterials).

To maintain a contemporary perspective, we restricted our research to English-language articles published in the 21st century. Our intention is to scrutinize and showcase how additive manufacturing and 3D modeling have emerged as game-changers in the cardiovascular field in the medical domain. In our review, we endeavor to include all the latest technological breakthroughs that have happened in the cardiovascular realm, primarily attributable to advancements in additive manufacturing and 3D modeling. We examine various treatments, procedures, and applications adopted in the cardiovascular field by clinical teams leveraging these cutting-edge technologies.

Based on insights gleaned from the articles reviewed, we further broadened and fine-tuned our search criteria. We incorporated new keywords, as stated in the three sections, to compile additional evidence and delve deeper into the subject matter.

## 3D Printing Technology in Cardiovascular Applications

Creating a 3D model involves sequential steps of diagnostic image acquisition, digital modeling, and 3D printing [23]. The first step in the 3D printing process is to obtain accurate solid digital representations of the subject that consist of multiple layered slices from computed tomography, magnetic resonance imaging, or 3D transthoracic or transesophageal echocardiography followed by image segmentation. The final step is the actual printing, and in this context, it is important to identify appropriate technologies and materials in consideration of the end use, and to consider the requirements of complexity, durability, and resolution of the model. Potential applications used in cardiac cases studies (as shown in Table 1) are stereolithography (or pool polymerization), powder layer casting (or selective laser sintering), material extrusion (or fused fiber filament/fused deposition modeling), and material jetting (or polyjet printing).

**Table 1:** Material and 3D printing technology used in case studies discussed in the review paper.

Study/references	Material	Printer/printing technology	Clinical tissue/organ	Year
Valverde et al. [24]	Translucent Polylactic Acid Polymer	Fused Deposition Modeling	Heart model with subaortic ventricular septal defect (VSD) and severe pulmonary stenosis	2015
Lee et al. [25]	2 Material: (i) Fused Filament fabrication in thermoplastic Polyurethane (TPU) and (ii) Combination of Magma H Line Photopolymer Resin and Monocure Flex100 Rapid Resin	2 Printer: (i) Ultimaker 2 Extended and (ii) Anycubic Photon S – Liquid Crystal Display (LCD)-based Stereolithography (SLA)	Three Heart Models – (i) 7-month-old boy with Ventricular Septal Defect; (ii) 17-Month-old boy with Double-Outlet Right Ventricular; (iii) 3-month-old girl with Tetralogy of Fallot	2021
Biglino et al. [26]	TangoPlus FullCure 930	Polyjet Printing Technology	Descending Aorta anatomy – Vertical and horizontal orientations	2013
Schmauss et al. [27]	–	Stereolithography Technology	Aorta including the aneurysm	2014
Ho et al. [28]	Strong Flexible plastic material	Selective Laser Sintering Technology	Aortic Aneurysm and Aortic Dissection	2017
Rynio et al. [29]	Clear resin	Stereolithography Technology	Aortic Arch	2018
He et al. [30]	ZR80 photoreactive resin polylactic acid	Stereolithography (SLA)	Patients heart model with Atrial Septal Defects	2019
Harb et al. [31]	TangoPlus for soft tissue and VeroWhite Material for calcification	Polyjet Printing Technology (Stratasys Connex 250 Printer)	Aortic Valve	2018
Vukicevic et al. [32]	TangoPlus	Polyjet Printing Technology	Mitral Valve	2017
Mashari et al. [33]	Silicone	Fused Deposition Modeling (MakerBot Replicator 2)	Mitral Valve	2016
Melchiorri et al. [34]	Poly(Propylene Fumarate) PPF Polymer	Digital Light Processing Stereolithography Technology	Vascular Graft	2016
Dominguez-Robles et al. [35]	Polycaprolactone Polymer	Fused Deposition Modeling (Bioscaffolder 3.2)	Vascular Graft	2021
Dominguez-Robles et al. [36]	Combination of Polycaprolactone Polymer and Acetylsalicylic Acid	Extrusion-Based 3D Printing	Vascular Graft	2021

Stereolithography (SLA) and powder layer casting technologies use lasers to fuse multiple layers of printed material. In stereolithography, an ultraviolet laser is used to harden the surface layer of liquid photopolymer resin, while in powder layer casting, the laser heats and melts each layer of the powder printing material without the need for a supporting structure. While there is a growing selection of photoreactive materials that can be used in stereolithography (SLA), it is important to note that the technology is still constrained by significant limitations, specifically, SLA is currently limited to the use of a single resin at a time when creating the final model [37]. In addition, SLA materials are stiff due to a high level of cross-linking, which limits their value for soft tissue mimicry. SLA offers spatial resolutions that are higher than those of most imaging modalities [38], this is not an intrinsic limitation, but is related to the need to avoid tissue damage from the penetrating radiations used in imaging.

Material extrusion and material jet technologies use a nozzle or jet to apply a liquefied printing material, which then solidifies to form a new layer [39]. Material extrusion technology creates 3D models by extruding thermoplastic materials or bio-ink filaments layer by layer. Material jetting printers produce 3D models by injecting thin layers of photopolymers that harden rapidly under the influence of ultraviolet radiation. This technology allows the combination of multiple materials and colors in the same print [40]. Polyjet also offers the possibility of processing photopolymer objects with a variety of physical and mechanical properties by varying materials such as those available from Stratasys—Tango™ (soft and rubbery), Agilus30™ (withstands repeated flexing), and Vero™ (rigid with a smooth surface). Thus it is possible to provide standardized material properties and to yield realistic haptic feedback [41] and the mechanical properties of valve leaflets that are required to simulate physiological aortic valve gradients [42]. Note that post-processing may also be required after printing is complete, typically these operations are cleaning to remove residue and support materials and sanding if smooth surfaces are required [40].

## Applications in Cardiovascular

**Fig. 1:**
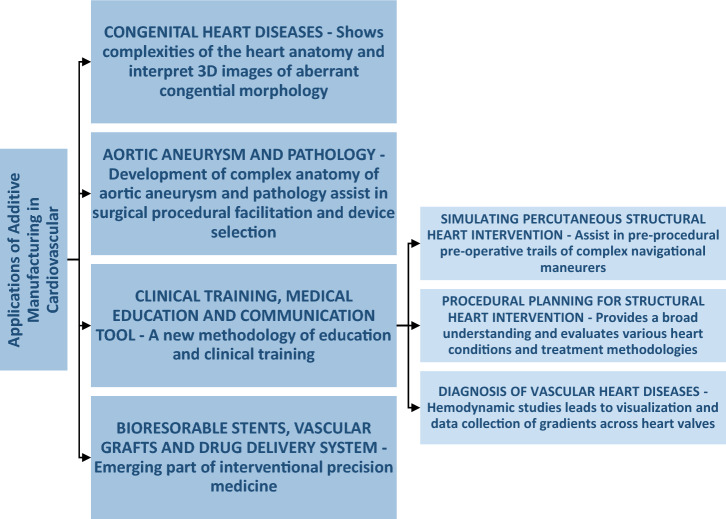
The board range of applications of additive manufacturing in cardiovascular has been divided into SIX major categories in this study

3D printed models have proven useful for the pre-operative rendering of target anatomy for various congenital heart diseases and structural heart interventions. Such models have also been used to demonstrate suitable valve repairs, to evaluate internal cardiac defects, vascular anatomy, selection of cardiac procedures for cardiac tumors, and other complex heart interventions. Figure 1 indicates the range of applications of additive manufacturing in cardiovascular studies.

### Congenital Heart Disease

Certain heart conditions, especially congenital heart disease in both adults and children, require a detailed preprocedural study of the procedure and an in-depth understanding of the depth and spatial information of the heart [24]. Currently, available technology does provide 2-Dimensional and 3-Dimensional imaging capabilities but creating 3-Dimensional models with the exact geometry of the heart with spatial characteristics helps to support a beneficial surgical outcome by allowing a in-depth study, analysis, and visibility of the heart condition and to assist in planning a successful heart procedure or surgery [43].

Congenital heart disease is one of the most common cardiac problems found in children and fortunately, technological advancement in the medical field means that most such children survive to adulthood, but the surgery and procedures can be extremely complex. Precise knowledge of cardiac anatomy is the starting point for initial assessment, follow-up diagnosis, and further therapy [44]. 2-Dimensional images have their limitations in showing the complexities of the heart anatomy and interpreting 3-Dimensional images of aberrant congenital morphology can be difficult, especially post cardiac surgeries due to the complex intracardiac connections which cannot be observed through 3-Dimensional visualization. An ideal solution to such complex anatomical procedures would be to create a patient-specific 3-Dimensional model of the malformed cardiac and aorta [45].

**Fig. 2:**
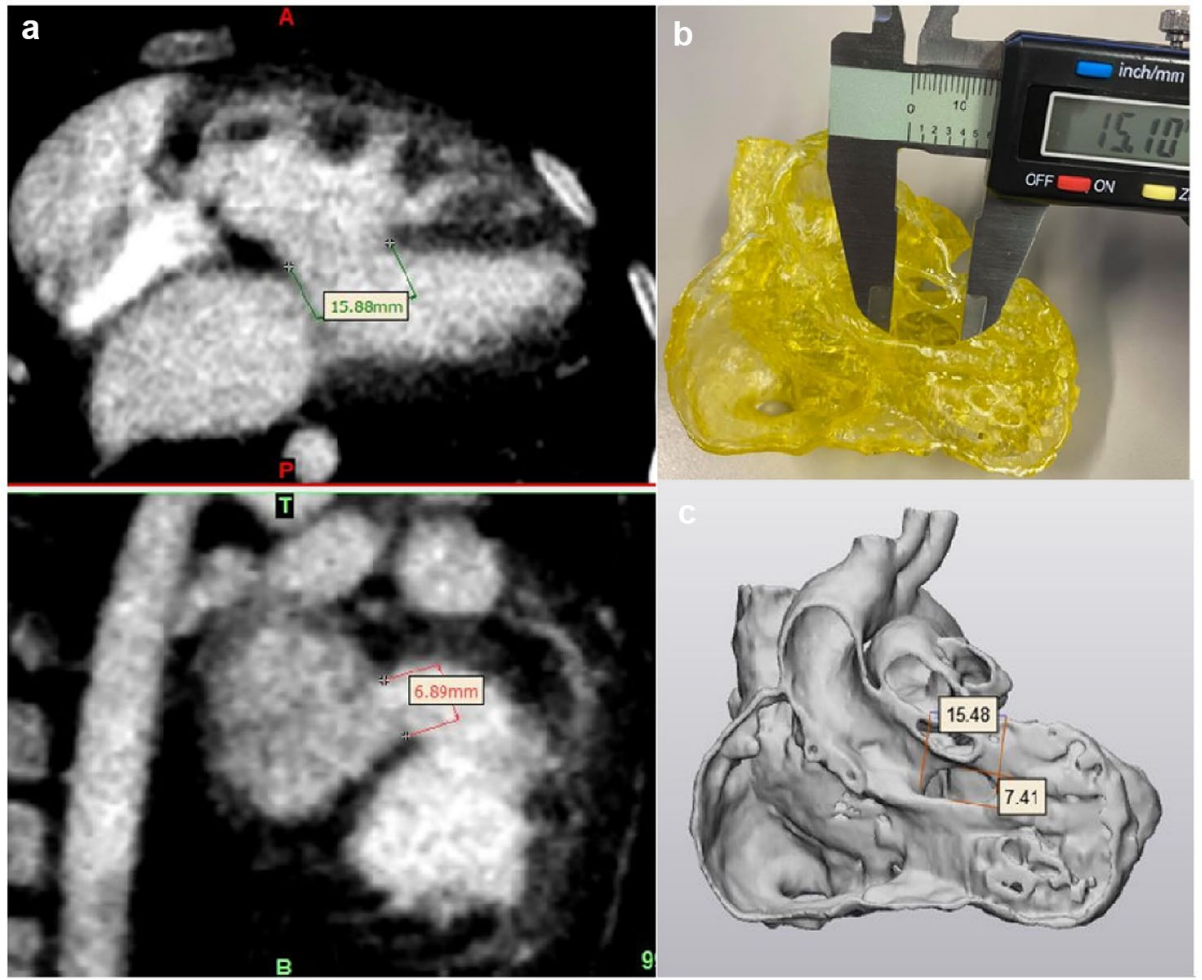
3D model accuracy evaluation of Ventricular Septal Defect (VSD). **a** Coronal and Sagittal views of CT Imaging Data. **b** Measurement of the VSD in the 3D printed model using a digital caliper. **c** STL file measurement of the VSD in 3-Matic. The 3D model assessment shows the correlation measurement of VSD on CT image, 3D Model, and STL file. The study shows that the measurements of the 3D models have a strong correlation (*r* = 0.99) and excellent reliability (Intraclass correlation coefficient = 0.97) compared to the original CT images, CT images of the 3D printed models, STL files, and 3D printed congenital heart disease models [25].

Current advancements in medicine and additive manufacturing have led to the development of highly accurate 3D printed models that replicate the cardiac anatomy and defects to assist in both presurgical planning and the simulation of congenital heart disease. One of the studies led by Lee et al. developed 3D models with strong correlation and excellent reliability compared with the original CT images that are shown in Fig. 2 [25].

### Aortic Aneurysm and Pathology

A common access point for endovascular treatment is the femoral artery, often catheters and medical treatment devices are directed via the aorta via the cervical arteries to the cerebral arteries and the site of the treatments. Typically, the training for these kinds of catheter-based endovascular treatments for vascular diseases uses animals [46], but there are certain ethical aspects to using animals for testing. Also, the vascular anatomy of a pig cannot be completely associated with that of a human reducing the overall effectiveness of the training. In addition, certain vascular curves seen in elderly patients cannot be well correlated to animal models [47]. Creating a modular aortic model using AM techniques with patient-specific anatomy can assist in understanding the complexity and help with the detailed planning of the procedure [48].

Early developments of physical Aortic model fabrication were recorded by Markl et al. [49] using MRI imaging where they successfully constructed the entire thoracic aorta and its main branches. Later, Sulaiman et al. [50] were successful in creating a 3D model patient aortic aneurysm to test for stent development and endovascular procedural simulation. A few years later, Biglino et al. [26] developed a descending aorta for in vitro studies and device testing. Schmauss et al. developed an aortic arch for preprocedural planning of a complex arteriosclerotic aneurysm patient surgery [27].

**Fig. 3:**
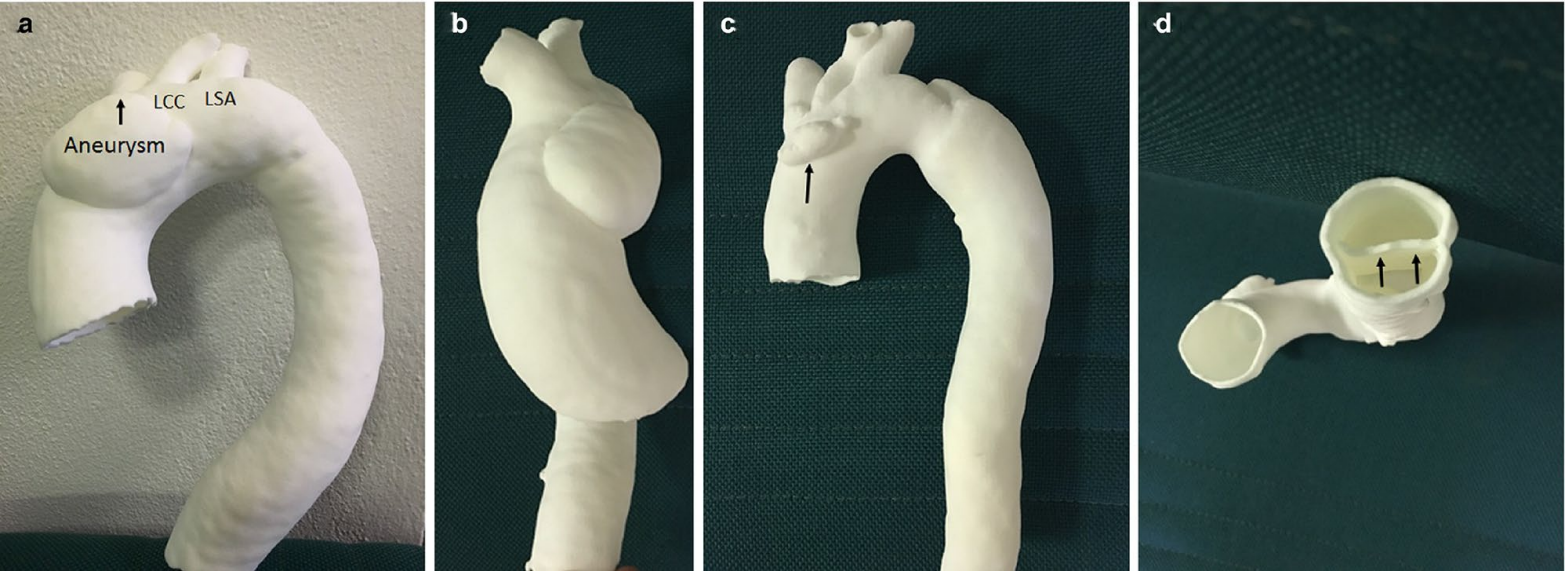
3D printed models generated from cardiac CT images which shows the Aortic Aneurysm structure from different angles. **a** Model shows the aortic aneurysm in relation to the three arterial branches starting from the aortic arch, namely left subclavian artery, left common carotid artery and innominate artery (arrow). **b** Lateral view showing the aneurysm. **c** Anterior view with artifact (arrow) in the aortic arch due to image post-processing. **d** Caudocranial view of aortic dissection showing intimal flap (arrows) [28].

Several recent studies have described developing the complex anatomy of aortic aneurysms and aortic pathology for surgical procedural facilitation and device selection to improve the accuracy and quality of treatment. As shown in Fig. 3, there are promising results reproducing 3D models depicting aortic aneurysm and aortic dissection by Ho et al. [28]. There is another case study where a 3-dimensional aortic arch was printed to facilitate accurate placement of openings in a graft [29]. Latterly, 3D models have been developed to study the fluid dynamics of blood flow in aortic aneurysms. A study showed conducting computational fluid dynamics modeling of abdominal aortic aneurysms indicating that this technique is a promising tool to gain clinical insights, assisting in understanding the biomechanics of the aneurysm and predicting the rupture potential of the abdominal aortic aneurysm [51].

Cardiovascular additive manufacturing has assisted in finding alternative solutions to aortic root replacement for patients with Marfan syndrome, Loeys–Dietz syndrome, and aortic root aneurysms [52]. 3D modeling of the patient’s aorta, dubbed Personalized External Aortic Root Support (PEARS) is a pre-emptive operation performed to halt aortic root expansion and maintain aortic valve function in Marfan Syndrome [53]. Conventional treatment would have taken the path of replacing the ascending aorta with a prosthesis and patients with valve disease would need to either replace the aortic root or deploy a valve-sparing root replacement (VSRR) [54]. PEARS is an approach that does not require opening the ascending aorta. A 3-dimensional copy of the patient’s aorta is created, and a soft polyester mesh sleeve of the same shape and size is individually formed on the implantation around the patient’s aorta, and it is then incorporated into the aortic wall [52]. As the aortic valve and the endothelium are preserved, no complications of the type that might occur from the Bentall procedure or VSRR arise [55].

**Fig. 4:**
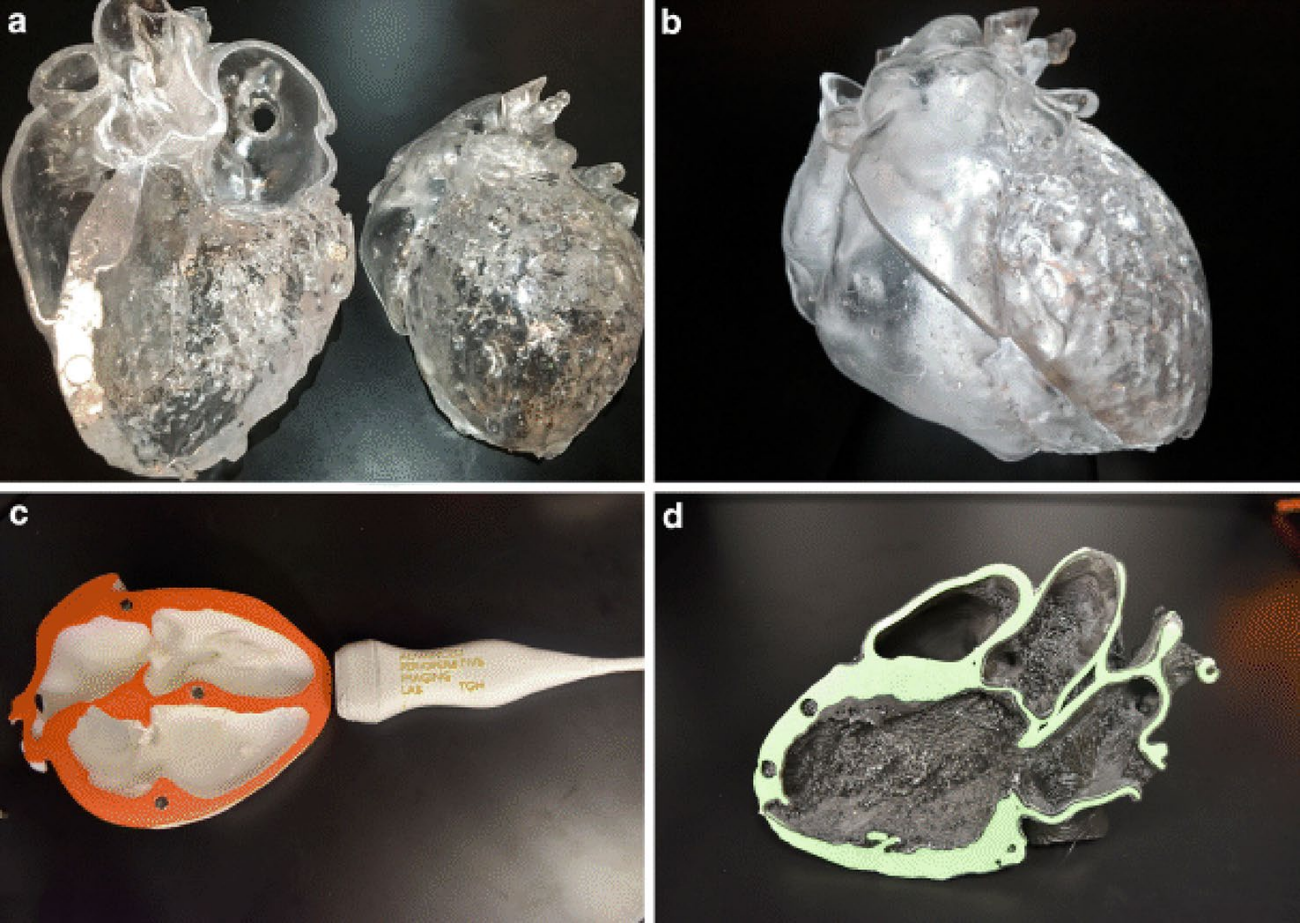
3D printing models lets the progress of precise life-like educational tools to demonstrate complex cardiovascular anatomy and pathology. **a**, **b** SLA transparent full heart model (normal anatomy) **c**, **d** Fused Deposition models illustrating standard transthoracic echocardiographic 2-Dimensional views (**c** apical four chamber view; **d** parasternal long axis) [61]

In the modeling aortic aneurysm pathology, most studies mention the need for accurate replication of internal complex aortic details. The high-resolution 3D modeling of internal components of the aorta will assist in making in-depth studies of the biomechanical and fluid dynamics properties of the cardiac tissues which should have a significant impact on future clinical treatments for patients with complex cardiac cases (Fig. 4).

### Clinical Training, Medical Educational, and Communication Tools

The Society for Cardiovascular Angiography and Interventions (SCAI) recognizes that medical simulators will take a greater role in cardiovascular training and retaining cardiovascular certifications [56]. Several cardiovascular societies, including the Cardiovascular and Interventional Radiological Society of Europe (CIRSE), the Society for Cardiovascular Angioplasty and Interventions (SCAI), the Society of Interventional Radiology (SIR), and the Radiology Society of North America (RSNA) have joined forces to mandate the use of simulators for cardiovascular catheterization training and education [56, 57].

Critical care training for clinical staff, residents, and nurses, on patient-specific 3D models before complex congenital heart surgeries, has not only improved the outcomes of the surgery but also enhanced communication between the cardiologists and patients [58]. A study done in the UK on educating adult and pediatric cardiac clinical nurses showed that 3D models assisted in the appreciation of the overall anatomy (86%), spatial orientation (70%), and anatomical complexity of the treatment (66%) [59].

**Fig. 5:**
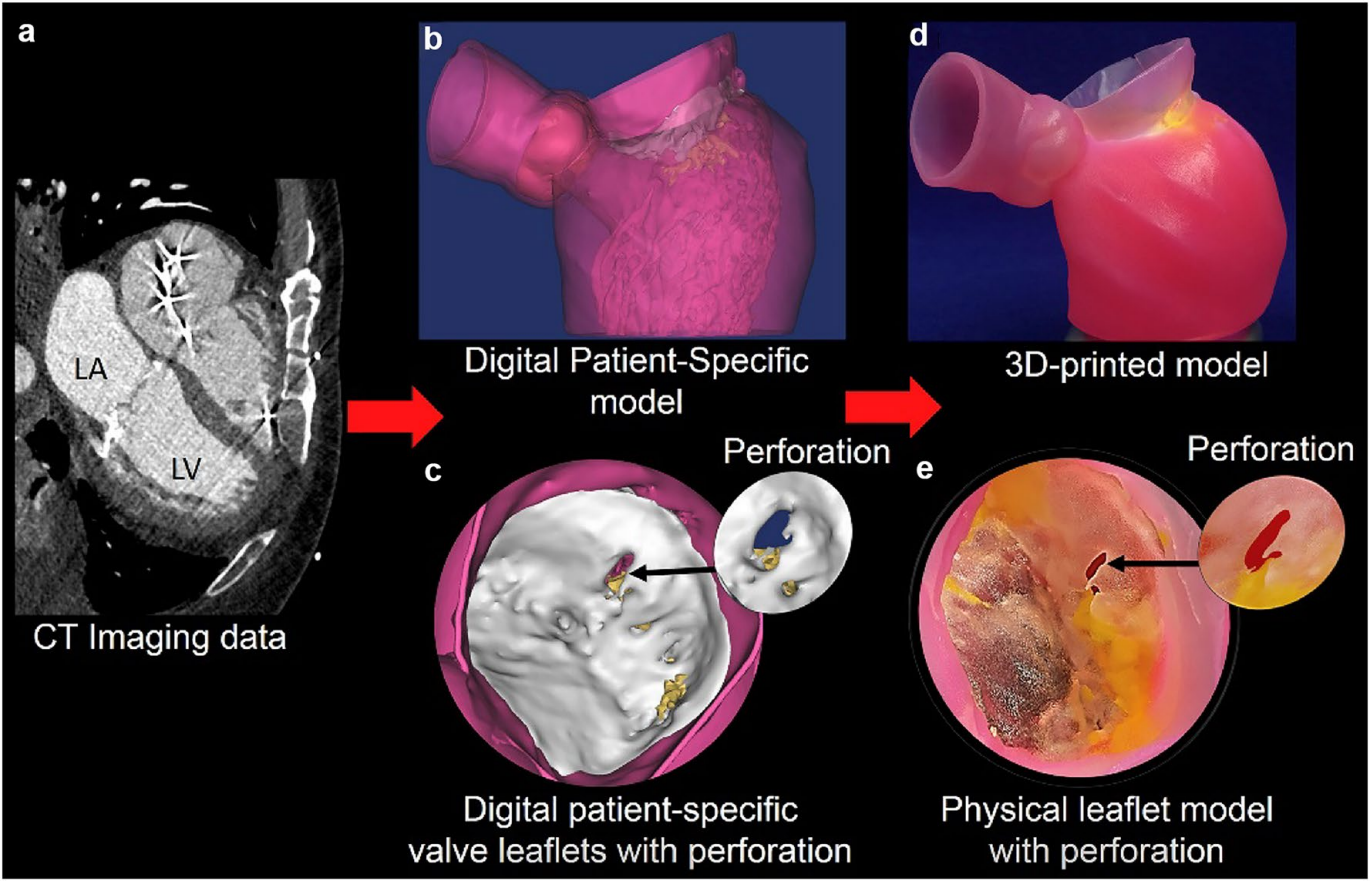
3D printing of Mitral Value geometry with regional calcium deposition (yellow) and Pathology shown in model E in the above figure. **a** CT Image **b** Digital patient-specific model **c** Digital patient-specific valve leaflets with perforation **d** 3D printed model **e** Physical leaflet model with perforation [84]

The current method of teaching congenital heart diseases to students is instructor-based using tools and limited practical techniques. Additive manufacturing and 3D printing can improve education by creating high-fidelity synthetic heart models with complex heart issues and applying these models to a novel, stimulation-based education program for premedical and medical students [60]. Figure 5 shows models of accurate and life-like heart models which can be used to illustrate complex cardiovascular anatomy and pathology [61]. Students that used 3D models for training were more receptive and this was reflected in higher student satisfaction scores and higher retention rates than those that did not use 3D models [62].

#### Simulating Percutaneous Structural Heart Intervention

One of the first 3D image fusion-guided transcatheter repairs of a sinus venous atrial septal defect was carried out on a patient by Thakkar et al. 2018 with the assistance of 3D modeling. A 3D model was developed using a tissue-mimicking rubber-like material from post-CT angiography image segmentation. The stent graft was positioned and then implanted into the model using fluoroscopy and post-simulation demonstrated successful nature of the exclusion. The model was demonstrated to the patient to obtain well-informed consent for the surgery [63]. The paper illustrates several advantages of the procedure:

(i) Objectives and procedural for any complications were well defined.
(ii) Assisted in identifying the potential challenges for device implantation.
(iii) Mimicking the procedure on a 3D model assisted in better imaging techniques during the actual surgery for visualization of the anatomic target for device deployment in a quasi-3D environment.

Another study showed simulation using a 3D model-assisted cardiac catheterization allowed preprocedural pre-operative trials of the complex navigational maneuvers needed to advance catheters from the aorta to the left ventricle and to position the catheter tip between the two papillary muscles, close to the mitral annulus [64].

One critical study in pulmonary valve replacement was carried out by Phillips et al 2016. The majority of Fallot patients, a defect caused by a combination of four heart defects at birth, are not candidates for Transcatheter Pulmonary Valve Replacement (tPVR) due to the large irregularity of Dysfunctional Right Ventricular Outflow Tract (RVOT). A hybrid approach was used to remodel the RVOT in transannular patch patients using preprocedural 3D model simulations, to evaluate different strategies, and so to design individualized solutions for patients with different cardiac anatomies [65]. A similar approach was done on a 15-year-old patient with D-transposition of the great arteries with combined neo-pulmonary stenosis and regurgitation following arterial switch operation as a neonate and neo-pulmonary valvotomy [66].

#### Procedural Planning for Structural Heart Interventions

A case report was reported by Dakowski et al. where a clinically applicable heart modeling technology was used for the preparation of a percutaneous mitral annuloplasty using the Mitralign percutaneous annuloplasty system [64]. This technique of direct percutaneous mitral annuloplasty requires the advancement of a guiding catheter through the aorta, into the left ventricle and requires accurate positioning of the tip of the catheter between the papillary muscles near the mitral annulus. 3D modeling assisted in creating a procedural plan and optimizing potential device implantation.

This was not the first time that 3D printing has assisted in better planning of innovative heart interventions. Another intervention was reported by Forte et al 2018 where patient-specific 3D modeling was created to accurately access the anatomy of Sinus Venous Atrial Septal Defect (SVASD) and Partial Anomalous Pulmonary Venous Drainage (PAPVD) to develop a safe and clinically effective interventional catheterization treatment for 3 patients [67]. A similar successful case was carried out by the same team on five patients using a two-step simulation (3D printed models and invasive balloon interrogation) for procedural planning and case selection in the transcatheter correction of superior SVASD with PAPVD [68]. Later, five patients diagnosed with multiple Atrial Septal defects were treated in interventional therapy using three-dimensional printed models which aided completion of the procedures with no device embolization, procedure death, or pericardial tamponade [30]. Another similar procedure was carried out in Fuwai Hospital at the Chinese Academy of Medical Sciences using CT scans and 3D echocardiography to provide data for 3D heart modeling and to construct heart models to be used to determine the best occlusion program for 21 patients [69]. In a minority of cases a mild residual shunt was observed during post-operative follow-up. These results from the application of 3D modeling may overcome the current limitations of CT and MRI imaging to allow definition of the functional morphology of Ebstein’s anomaly, atrioventricular septal defects, and evaluation of mitral regurgitation. Table 2 shows the examples of Cardiovascular case studies used for Procedural Planning of Structural Heart Interventions.

**Table 2.** Examples of cardiovascular case studies used for procedural planning of structural heart interventions

Clinical condition/procedure	Description of use of 3D model	References
Heart with transposition of the great arteries, ventricular septal defect, and pulmonary stenosis	Cardiac model assisted surgeon to evaluate the location and dimension of ventricular septal defect as well as its relationship with aorta and pulmonary artery Assisted in reduced operative time and Morbi-mortality	Valverde et al. [24]
Resection of ventricular aneurysm and right ventricular tumor	3D printed Left ventricular aneurysm assisted reshaping the left ventricle ensuring enough left ventricle volume was accomplished 3D model usage during resection of ventricle aneurysm and malignant cardiac tumor facilitated surgical procedure through better planning and improved orientation	Jacobs et al. [43]
Percutaneous Mitral Annuloplasty	3D model developed using Multi-slice Computed Tomography (MSCT) 3D model used to create procedural plan to optimize potential device implantation Helpful tool for individualized planning of percutaneous structural interventions but future studies needed to warrant its role in preparing for percutaneous and surgical heart procedure	Dankowski et al. [64]
D-transportation of great arteries status post Mustard presents with dyspnea	3D model was developed using CT images and 1:1 model printed. Multiple catheter approaches to the pulmonary venous atrium were trailed on model. Once mode catheter access to pulmonary was selected, stent delivery was planned, and stent was deployed with model to visualize its position within stenosis and adjacent structures	Olivieri et al. [70]
Transcatheter Valve replacement (patient with aortic valve stenosis with severe calcification of ascending aorta)	Technique to fabricate life-like models to evaluate transcatheter aortic valve replacement Did not change the basic surgical plan, but assisted in learning the exact position of critical structures and anticipate difficulties which helped with reducing perioperative risk	Schmauss et al. [71]
Endovascular Interventional Planning (Multiple Asymptomatic Splenic Artery Aneurysms)	Create an anatomically accurate hollow vascular 3D model and test different catheters and stents Assisted in providing clear visualization of the surgery and select the appropriate procedure During procedure, best angiographical angles for optimal visualization were selected due to which repeat angiograms were avoided, saving both time and intravenous contrast	Itagaki et al. [72]
Complex obstruction at neoaortic to transverse arch and descending aortic junction following the neonatal modified Norwood-1 procedure for hypoplastic left heart syndrome	3D model created using CT images Model provided detailed insight to the anatomy and surgical approach and steps of operation simulated onto the model Pre-operative stimulation assisted in achieving relief obstruction without additional patching, and contributed to successful outcome and improved patient safety	Kiraly et al. [73]
Cardiac tumor	Assisted in therapeutic decision-making process by showing the exact position and infiltration of cardiac tumor into cardiac tissue where conventional imaging had limitations Provided vital structural insight to tumor size, location, and extension prior to complex cardiac surgeries	Schmauss et al. [74], Jabbari et al. [75]
Boy with double-outlet right ventricle underwent bidirectional Glenn anastomosis	3D model provided invaluable intracardiac spatial information for the biventricular repair	Farooqi et al. [76]
Patients with complex congenital heart disease like double-outlet right ventricular, criss-cross atrioventricular connections, and congenitally corrected transportation of great arteries with pulmonary atresia	Patients included had complex heart conditions with previously unresolved management decisions Models assisted in understanding the heart anatomy, identify specific technical challenges and assist in surgical planning Surgeries were executed as per the pre-operative planning using the 3D models and the models assisted in decision-making, planning and safe execution of complex heart surgeries Assisted in anticipating all complex technical challenges of the surgery	Kappanayil et al. [77]
Occlusion of an Ascending Aortic Pseudoaneurysm	Successful occlusion via Intraoperative Echocardiography and 3D printed Model	Li et al. [78]

#### Advanced Modeling of Valvular Heart Disorders

The procedural planning of appropriate surgical cases typically now uses 3D modeling as assistive technology in the diagnosis of several cardiac-related issues. 3D modeling has assisted in defining the aortic anatomy in aortic stenosis cases including those affecting the aortic valve area, root morphology, calcium distribution, and distance to coronary arteries [79, 80].

3D printed models have evolved from the first-generation modeling used for initial training of the Transcatheter Aortic Valve Replacement (TAVR) procedure to multi-material printed models to mimic tissue properties for ex-vivo visualization of valve implementation [81]. Recently, the functional comparison of the hemodynamic features of native and implanted valves was successfully carried out using 3D modeling [82]. Another group used 3D modeling to re-create a confirmed case of coronary artery occlusion by building a patient-specific ex-vivo physiological flow model using a pulse duplicator and quantifying fractional flow reserve at transcatheter valves in various annular positions [83].

One of the first patient-specific heart 3D models was made to aid in the planning of a percutaneous Mitral Valve repair where the 3D model demonstrated morphological details that assisted the team in the planning of catheter manipulation and device implantation [64]. In vitro simulation of percutaneous Mitral Valve repair was carried out by Little et al 2016 who reconstructed the entire mitral valve apparatus with flexible materials to visualize leaflet deformation, and thence device selection, and implantation—see Fig. 4 [84]. Recently, patient-specific modeling using a mitral valve simulator (MVS) pulse duplicator was proposed as part of the routine workflow for evaluating transcatheter mitral valve interventions [85].

Of all the heart valves, the tricuspid valves have the least reports in the literature related to 3D modeling. One limitation is concomitant renal failure, which precludes the administration of intravenous contrast in many patients [86]. A recent report used a combination of non-contrast CT, cardiac MRI, and/or transesophageal echocardiography (TEE) to generate data sets for contrast intolerant patients, but the anatomical details of the valve were not detectable [87]. 3D TEE has also been used to create annulus and leaflet models but is unlikely to adequately reproduce the fine anatomical details of the valve, including seals, rims, or sub-valves [88]. However, Vukicevic et al. described the use of patient-specific 3D printing and 3D TEE imaging in the preprocedural planning of percutaneous tricuspid valve repairs [89].

One advance in cardiac diagnosis is the development of functional 3D models for hemodynamics studies across aortic valves which has great potential for diagnostic and preprocedural applications. Harb et al. 2018 developed a patient-specific 3D model of severe aortic stenosis with matching anatomical and hemodynamic properties including stroke volume. Models of this type can assist in the characterization of challenging cases where there is a discrepancy between aortic valve morphology, calculated aortic valve area, and estimated transaortic gradient. However, the study had limitations:

(i) the Valve is printed in only one phase of the cardiac cycle, typically mid-systole.
(ii) the number of parameters that impact aortic flow and gradient (coronary arteries origins and flow, systematic peripheral resistance) were not considered [31].

Certain functional 3D printing studies have been successfully carried out on the mitral valve for catheter-based structural interventions for hemodynamics studies and evaluation [32]. One study showed promising results for hemodynamics testing of patient-specific anatomy and mentioned possible future applications of visualization and data collection of gradients across the aortic valve [33]. The technology is involved and there is room for further improvement as the treatment is proving to be a successful tool for high-risk patients. Treatment standards and protocols need to be set to use the technology for normal conventional treatments to increase the quality of patient care, increase treatment accuracy, and reduce treatment complications. It is worth noting that 3D modeling has not yet been widely adopted to replace the aortic valve, mitral valve, tricuspid valve, or any heart valve disease but the techniques hold a promising future. 3D modeling with tissue-mimicking materials to perform in vitro simulations can improve decision-making regarding both valve sizing and positioning in these higher-risk patients and help reduce cardiac surgical complications.

### Bioresorbable Stents, Vascular Grafts, and Drug Delivery Systems

Bioresorbable stents (BRS) or Bio-absorbable vascular stents (BVS) are the most recent advance in stent technology having properties of biosorption, mechanical flexibility [90], and the ability to clear the body of any thrombogenic foreign matter. This overcomes some major limitations of currently available permanent stents [91]. BRS also improves the long-term patency rates by providing support while the artery heals, but no longer [92]. Other properties of the stents include minimal toxicity to the human body, degradation rates relating to the rate of recovery of vascular tissues, and the induction of rapid endothelialization to restore the function of the vascular tissue [93].

The additive manufacturing approach for BRS manufacturing has gained popularity in the last few years because of the resulting properties [94], economic benefits [95], general feasibility [96], and reasons relating to patient-specific model creation [97]. One of the first additive manufacturing processes of creating a BRS and studying its effects on animals was carried out by Park et al. They fabricated a 3D printed drug-coated BVS for animals and from this work suggested a new approach for treating coronary thrombosis by providing a novel and promising scaffold for stent technology [98]. Another promising animal study on mouse venous systems was used to develop techniques and materials for vascular tissue engineering for cardiovascular application [34]. The analysis of results showed that the designs had adequate mechanical properties which could support vascular tissue growth both in in vitro and in vivo.

**Fig. 6:**
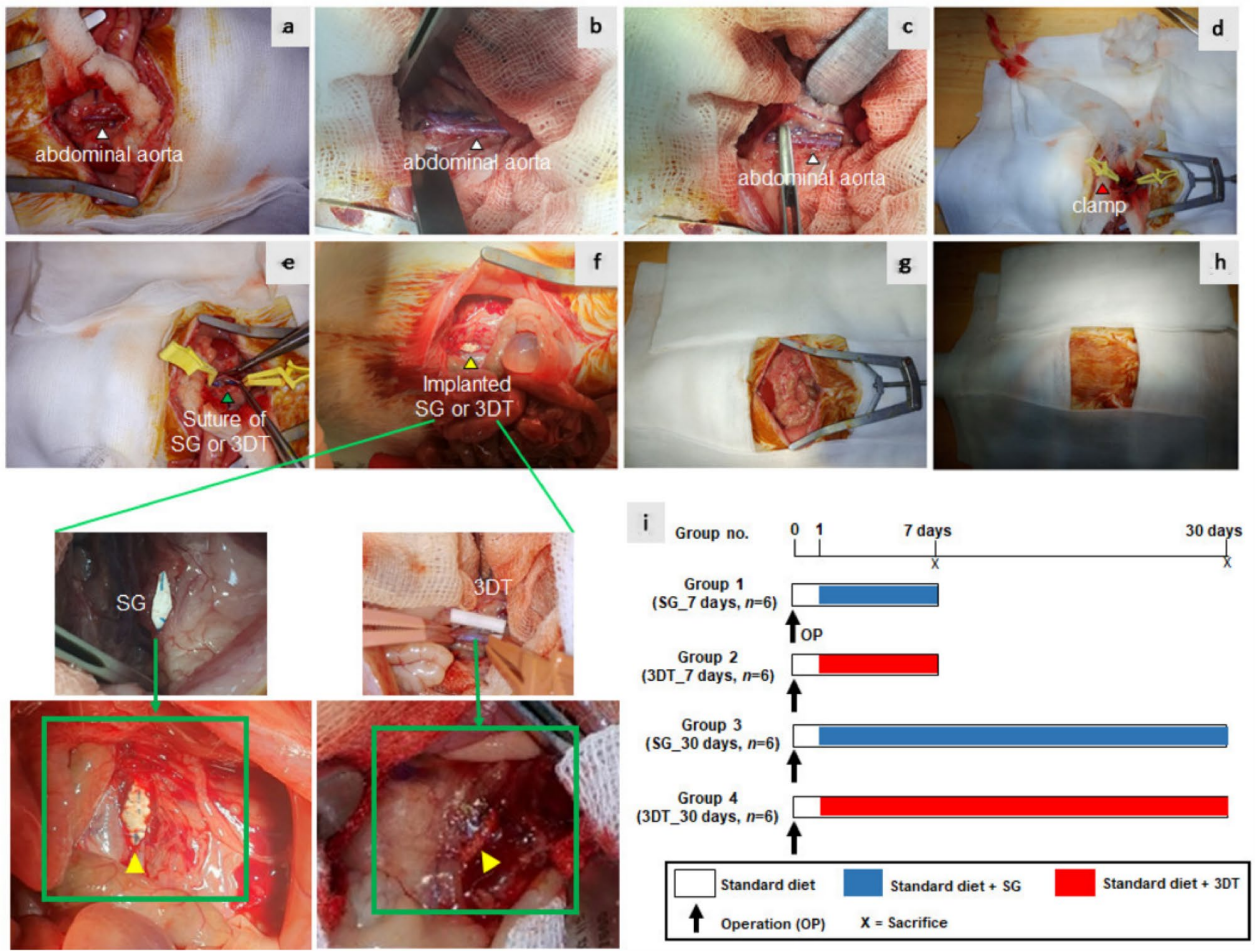
Illustrates the experimental design of In vivo implantation of vascular patches using standard grafts (SG) or 3D templated vascular grafts (3DT) using rat abdominal aorta model **a**–**c** White arrows indicate abdominal aorta; **d** red arrow indicates clamp; **e** green arrow indicates suture with SG or 3DT vascular patch; **f** yellow arrow indicates implanted SG or 3DT vascular patch; **g**, **h** the wound was closed layer by layer; **i** experimental design [101]

Revascularization is a process of restoration of tissue perfusion to remediate cardiovascular disease and to restore vascular tissues to reduce the risk of heart failure [99]. Thick, durable vascular tissue structures can be designed, printed, and cultured in vitro, offering a promising alternative to traditional vascularization medicine [100]. Synthetic vascular grafts were developed with properties of antithrombosis, neointimal hyperplasia inhibition, and fast reendothelialization by creating 3D printed biodegradable vascular grafts with precise geometries for tailored revascularization procedures [35]. The grafts were extensively characterized and were evaluated for drug release characteristics, antiplatelet effects and cytocompatibility. The studies showed reduced platelet deposition, sustained and linear drug release, and the provision of a beneficial environment for cellular attachment, viability, and growth [35]. A synthetic 3D templated vascular graft was developed and was tested in vivo on rats by Sohn. The grafts are not yet superior to commercially available synthetic grafts but are a preliminary analysis for the possibility of using 3D templated (3DT) vascular graft in clinical applications, as they showed a reduced risk of thrombogenesis and intimal hyperplasia and facilitated endothelial cell activation [101]. Figure 6 illustrates the various surgical steps and indicates the various significant anatomical structures involved in the procedure.

Our systematic literature search showed that various studies have analyzed and developed materials for BRS giving the required properties and made using additive manufacturing techniques [42, 94, 98, 102] but very limited work, except for a few animal studies was found that actually implemented these materials for clinical cardiovascular use. The real application of 3D fabricated stents is limited today due to material, machine, and manufacturing constraints but there is ongoing research and development for future stent technology development using additive manufacturing.

A Polymeric drug delivery system is a device that facilitates the introduction of a therapeutic substance into the body and is a technology that has significantly developed in the last two decades [103]. However, the introduction of biodegradable polymers and additive manufacturing in such drug delivery systems is yet unexplored for cardiovascular applications [36]. Both the revascularization studies mentioned above (see “bioabsorbable stents and vascular grafts”), applied these drug delivery methods and the studies did show sustained, linear drug release rates [35, 36].

## Challenges and Limitations

One of the limitations mentioned in most of the studies was the imprecision of 3D models due to the poor initial resolution of the 3D images. Medical images need to be isotropic with a high spatial resolution to provide rich detail and high contrast to distinguish the adjacent structures. Low resolution and the presence of artifacts can produce false diagnoses [104]. There has been evidence that some artifacts that affected the acquisition parameters and resolution of the image led to final volume errors [105]. A systematic review on congenital heart disease mentioned that the two main reasons for limitations of printed models are first the result of image acquisition technique limitations that result in poor image resolution [106].

The second major limitation discussed is the re-creation of soft tissues and vascular structures which consists of complex multicellular arrangement, involving a controlled combination of vascularization, and mechanical, biological, and electrical properties. Additional techniques such as biomechanical stimulation and electrical stimulation are used to improve 2-Dimensional cultures, but they lack the crucial anatomical and physiological properties of cardiovascular micro-environments and thereby fail to provide an effective strategy to generate viable cardiac tissue and are thus not suitable for developing disease and drug screening platforms [107, 108].

In addition, the combined development of printing technologies and biomaterials are giving faster, more efficient, and more cost-effective results. If accompanied by better resolution of 3D images from imaging modalities such as CT and MRI this will enable the development of high-definition 3D modeling for not just cardiovascular applications but also for other healthcare and pharmaceutic applications.

## Future Directions

Clinical requests for a 3D printed model before imaging acquisition can result in the use of separate CT and MRI protocols to improve spatial resolution, spatial fidelity signal-to-noise ratio, and the contrast-to-noise ratio resulting in a better printed model [109]. Technological advancements in imaging modalities such as CT and MRI and imaging acquisitions developments such as time-resolved or motion incentive radial data acquisition, and deep-learning based image reconstruction can also assist in the formation of high-resolution 3D and 4-Dimensional imaging which will assist in printing precise and highly detailed 3D models [110].

Personalized cardiovascular 3D printed prostheses are not currently available but are imminent [111]. Progress must continue toward the development of implantable, 3D printed models which can be patient-specific for use in maxillofacial and orthopedic repairs [112]. Unique custom-made medical devices and implants which are patient-specific can bring better outcomes for patients with abnormal anatomy, complex structures, or neoplasms for which challenge conventional techniques [113].

Heart-on-a-chip technology and engineering innovations to re-create physiological conditions of organs are emerging as popular tools in drug delivery because of their potential for automated control of culture conditions (medium oxygenation, pH level, temperature control, etc.), application for heart stimulations (electrical or mechanical), and continuous monitoring and measurements of physiological responses—all of which are enabled by the application of built-in readouts, sensors, and electrodes [114]. The convergence of additive manufacture technology and heart-on-a-chip technology can offer an efficient route in developing complex organ and tissue structures with precise and specific physiological, mechanical, and patient-unique properties which can be used to create automated and high-throughput platforms for various other applications such as toxicity evaluation and drug delivery systems development [115].

One of the studies on 3D bioprinting for cardiovascular regeneration mentions the following challenges which need to be addressed to assist in the development of a whole functional cardiovascular system:

(i) Obtaining sufficient human-derived cardiovascular cells to achieve physiological distribution of associated cells.
(ii) Development and optimization of bio-inks for cell viability, phenotypes, and functionality.
(iii) Development of better bioprinters to improve speed and resolution.
(iv) Creation of perusable vascular networks in thick constructs for improving the functional and biomechanical integration with the host cardiovascular system.
(v) Balance of structural stability, degradation rates, flexibility, and proliferation space of printed myocardial cells [116].

## Conclusion

Several significant advantages resulting from the use of additive manufacturing have been reported:

(i) Personalized patient care with precise preprocedural planning.
(ii) Reduction in intraprocedural guesswork regarding catheter and device selection, thus improving patient safety.
(iii) Improved operator confidence as 3D modeling assists in viewing models in all angles and views.
(iv) Simulation and maneuvering of 3D modeling assists in gathering additional information along with traditional imaging.
(v) Assisting operators to foresee intraoperative complications and plan better [117].

3D models are currently derived from CT and MRI images; it is desirable that 3D modeling should overcome the current limitations of CT and MRI imaging to better define the functional morphology of Ebstein’s anomaly, atrioventricular septal defects, and for the evaluation of mitral regurgitation [117, 118]. Several studies have been carried out to compare diagnosis using advanced imaging technologies for cardiovascular treatments with those done using 3D modeling, which show that 3D printing, and additive manufacturing applications offer significant advantages. A study was carried out working with endovascular operators by Tam et al 2016 where the endovascular operators were asked to review results from CT angiography and make a management plan. The operators were later asked to re-evaluate their plan using an equivalent 3D model. 20% of the management plans changed when presented on revaluation using 3D models and of the plans that didn’t change, a 43% level of increase in confidence was observed, hence, demonstrating a quantified improvement from the use of 3D models to assist in planning endovascular aneurysm repair cases [119].

The insight provided by additive manufacturing has the potential to increase the efficiency of cardiac procedures by improving conventional and surgical planning as well as reducing radiation exposure [120]. Further integration and technological advancement of other imaging technologies in combination with 3D printing can provide a single platform for virtual dynamic 3D modeling and this hybrid modeling technique can drive the future of cardiac imaging [121].

For in vitro device testing and procedural stimulation, 3D printed cardiovascular models mimic the appearance, mechanical behavior, as well as the dynamic behavior of the target organ through the cardiac cycle. Challenges still exist in finding the perfect match for the biologic tissues due to the inability of currently available processable materials to precisely mimic the non-linear and anisotropic behaviors of biologic tissues [122]. In the last decade, three-dimensional models have been used in cardiovascular applications to enhance the understanding of complex congenital heart disease and thence to assist in surgical planning by simulating structural percutaneous interventions [104].

Additive manufacturing is an evolving field concurrent with industry 4.0 but significant application and advancement in medical device and cardiovascular applications has only begun in the last decade. For this reason, more technological research and studies need to be carried out in the field of additive manufacturing to develop a high-resolution printing technology and to develop 3D printable biomaterials which are non-thrombogenic. A comprehensive assessment of materials and their mechanical and physical properties along with process optimization and blood compatibility studies can assist in the rapid and beneficial commercialization of additive manufacturing technology for medical implants and general medical science. The technology meets the various requirements in the cardiovascular field due to its flexibility and suitability for a number of patient-specific applications. For these same reasons, this technology has the capability of tackling various challenges that cannot be achieved with current conventional manufacturing technologies. In the future, additive manufacturing may have the capability to print a working smart material 3D heart model. This type of working model includes an effective time dimension and so is known as a 4-Dimensional model. Developing this technology to grow inside a patient’s body and to respond to patient-specific conditions (so-called menotic materials) would open a whole another dimension of patient care and cardiac surgeries with the potential to revolutionize the fields of cardiology and general medicine.

## Abbreviations

3D: Three-dimensional
AI: Artificial intelligence
AM: Additive manufacturing
BRS: Bioresorbable stents
BVS: Bio-absorbable vascular stents
CT: Computed tomography
CVD: Cardiovascular diseases
FDM: Fused deposition modeling
MRI: Magnetic resonance imaging
PAPVD: Partial anomalous pulmonary venous drainage
PEARS: Personalized external aortic root support
tPVR: Transcatheter pulmonary valve replacement
RVOT: Right ventricular outflow tract
SLA: Stereolithography
STL: Standard triangle language
SVASD: Sinus venous atrial septal defect
TAVR: Transcatheter aortic valve replacement
TEE: Transesophageal echocardiogram
VSD: Ventricular septal defect
VSRR: Valve-sparing root replacement
